# Validation of the Multicultural Personality Questionnaire Short Form (MPQ-SF) for use in the context of international education

**DOI:** 10.1371/journal.pone.0244425

**Published:** 2020-12-28

**Authors:** Joep Hofhuis, Joran Jongerling, Karen I. Van der Zee, Jeroen Jansz

**Affiliations:** 1 Erasmus Research Center for Media, Communication, and Culture (ERMeCC), Erasmus University Rotterdam, Rotterdam, The Netherlands; 2 Erasmus School of Social and Behavioral Sciences (ESSB), Erasmus University Rotterdam, Rotterdam, The Netherlands; 3 Faculty of Social Sciences, VU University Amsterdam, Amsterdam, The Netherlands; Universitat de Valencia, SPAIN

## Abstract

The Multicultural Personality Questionnaire (MPQ) is one of the most widely used instruments for measuring individuals’ intercultural competences. The original version consists of 91 items, divided into five subscales, and has been shown to predict attitudes, behavior, and outcomes in a variety of intercultural contexts. Recently, a 40-item short form of the MPQ was developed (MPQ-SF), which may be particularly useful in settings in which time or survey space are limited, or where respondent drop-out is likely to occur. For example, the MPQ-SF would be a valuable tool for assessing longitudinal development of multicultural personality traits in training or educational settings. A prerequisite for such research is to establish measurement invariance of the MPQ-SF between different respondent groups, as well as across time points. Using a sample of students in an international university program (*n* = 519), the present study examines how the scales perform among male and female respondents, between students of Western and Non-Western background, and across two time points, five months apart. Based on our findings, we conclude that all five subscales of the MPQ-SF display sufficient measurement invariance to be reliably used in this and similar contexts, in comparative as well as longitudinal study designs.

## Introduction

Due to globalization and increased international mobility, it has become much more common for people to interact with others with different cultural backgrounds. Studies have shown that individuals may react differently to cultural diversity in their social environment, leading some to be better able to communicate effectively across cultural boundaries and adjust more successfully to intercultural situations [1–3]. A significant predictor of these intercultural competences is personality [4–6]. Through meta-analysis [7], it was established that domain specific personality traits, such as cultural empathy and cross-cultural self-efficacy, are better predictors of intercultural effectiveness than more general personality traits such as the Big Five [8]. Therefore, to predict how individuals behave in intercultural contexts, scholars have identified personality dimensions that are specific to the cross-cultural domain, leading to sets of traits that are together referred to as *multicultural personality* [9, 10].

### Multicultural Personality Questionnaire (MPQ)

The most commonly used multicultural personality framework is the Multicultural Personality Questionnaire [MPQ; 11–13]. The MPQ is anchored in organizational and cross-cultural psychology and was initially developed for assessing intercultural effectiveness of sojourners, such as international students and expatriates. Over the past two decades, its use has been extended to many other contexts, such as acculturation of migrants [14], intercultural training [15] and the job market [16]. Through a significant and growing number of studies, the MPQ has been revealed as one of the most robust instruments for assessing intercultural competence [17].

The full version of the MPQ consists of 91 items, divided into five subscales, each of which measures a separate multicultural personality trait [12]. *Cultural Empathy* (CE) refers to empathizing with the feelings, thoughts, and behaviors of individuals from a different culture; *Emotional Stability* (ES) reflects the individual’s ability to stay calm under novel and stressful conditions; *Flexibility* (FX) is the ability to switch easily from one behavioral strategy to another; *Openmindedness* (OP) refers to an open and unprejudiced attitude toward cultural differences; *Social Initiative* (SI) refers to a tendency to actively approach social situations, initiating communication rather than waiting and watching.

Earlier studies link MPQ dimensions to many intercultural outcomes, including sociocultural adjustment [18–20], acculturation strategies [14], effectiveness in intercultural interactions [15, 21], workplace discrimination [22], diversity beliefs [23], expatriates’ job satisfaction [24], and physical and mental health [25]. A significant proportion of the studies that utilize the MPQ have examined how its dimensions relate to behavior, attitudes, and outcomes of (international) students. For example, among university students in the Netherlands, the dimensions of cultural empathy, openmindedness and flexibility predicted motivations and intention to go abroad [11]. During the sojourn, all five dimensions have been reported to relate to mental health and subjective well-being of foreign students in the Netherlands [13], and emotional stability and social initiative contributed directly to international students’ adjustment in the United States [26]. Among Chinese and Malaysian students in New Zealand, cultural empathy and emotional stability appear to be strong predictors of socio-cultural adjustment [27], and among a sample of Singaporean undergraduates on international exchange, all five dimensions positively relate to socio-cultural adjustment, and all except flexibility appeared to enhance psychological adjustment [18]. Finally, two studies among multinational samples of students in British universities revealed that MPQ scores may be dynamic over time [28], and that intercultural group-work can lead to an increase in flexibility, as well as a decrease in openmindedness [29]. In sum, previous research shows that the five dimensions of the MPQ are relevant factors in shaping students’ effectiveness in the context of international education.

### Development of the Short Form (MPQ-SF)

In 2013, a 40-item short form of the instrument was developed [MPQ-SF; 30], which has been shown to display strong correlations with the original MPQ scales. Although the long version remains the instrument of choice for use in assessment and diagnostics, the short form may provide a reliable alternative in certain research contexts, specifically when time or space constraints play a role, or when respondent drop-out is more likely to occur.

The study that established the original development and validation of the short form [30] was conducted among a racially diverse sample of university students in the United States. It does not provide information on how the short scales perform among different groups of respondents. The first aim of the present study, therefore, is to further establish the validity of the MPQ-SF by examining between-group measurement invariance. Firstly, we will compare whether the subscales perform similarly between male and female respondents. Secondly, we will examine how it performs among individuals with different cultural backgrounds. This cross-cultural validation is particularly essential for future use of the instrument, since it is purposely designed for implementation in international and/or intercultural environments.

### Longitudinal development of multicultural personality traits

One of the major challenges in the research area of intercultural competence, is to examine how traits and skills develop over time. There is a growing interest in uncovering measurable effects of training and education on intercultural effectiveness [1], and as an extension of this question, if exposing oneself to an intercultural interactions may contribute to the development of MPQ dimensions. The MPQ was originally designed as a personality measure [11], implying that its traits are relatively stable over time. However, it has become apparent that individuals can indeed train intercultural competences through intercultural learning and experiences [31, 32], and may also increase their MPQ scores as a result [29].

A particularly interesting area of exploration is the development of multicultural personality among university students in international university programs. Many institutes of higher education around the world emphasize internationalization as one of their key features, which has resulted in a profound increase of the number of students completing (part of) their education abroad [33]. The long-term effects of these processes on students’ multicultural personality remain poorly understood [34].

In order to tease out how MPQ dimensions change as a result of international education, scholars need to employ longitudinal study designs, and compare scores at different points in time. Because of the importance of reducing respondent drop-out, the MPQ-SF may be a particularly useful instrument for this type of research. However, to be able to use it as an instrument for tracking longitudinal development, it is important to first establish reliability of its subscales across time points. Earlier studies have reported on changes in average (long scale) MPQ scores between different measurements [18, 28, 29], but longitudinal measurement invariance has not been examined for either version of the instrument. As such, the second aim of the present study is to establish longitudinal validation of the subscales of the MPQ-SF in this particular context.

## Methods

### Sample and procedure

The study was approved by the Ethics Review Board of the Erasmus School of History, Communication, and Culture (ESHCC) at Erasmus University Rotterdam. Written informed consent was obtained from all respondents. The sample for this study consisted of two cohorts of first-year students, of an international English-language bachelor program, at a major research university in the Netherlands. Students were invited to complete the Multicultural Personality Questionnaire–Short Form [30] at two different time points, five months apart. The first invitation was sent after the students were notified of admission to the program, four months before the start of the academic year (T1—May). The second invitation was sent one month after the start of the program (T2—October). This approach allowed us to compare how the MPQ-SF performed in two different context, just before and after a major life event.

All questionnaires and related communication were in English. In order to apply for the program in question, non-native speakers of English were required to demonstrate proof of proficiency in English through a TOEFL, IELTS or Cambridge certificate. As such, we assume all participants were able to understand and complete our questionnaires. An informed consent form was included at the start of each questionnaire, asking participants to confirm they had read and understood the conditions of the study, and agreed to participate voluntarily. No compensation was given for participating.

The recruitment of respondents was conducted in two consecutive years (2017–2018), with identical procedures for each cohort. At T1, the digital invitation to complete the questionnaire was sent by e-mail to all students who had been admitted to the program. Across the two years, a total of 658 respondents were invited to participate in the first wave, of whom 456 (69.3%) responded. At T2, students who ultimately decided not to enroll in the program were removed from the mailing list. Across the two years, a total of 480 active and enrolled students were sent the invitation to participate in the second wave, and were given time in class to complete the questionnaire. Nearly all (453; 94.4%) responded. The final sample used in this study consisted of 519 respondents (60.5% female, 14.5% male, 25.0% other or unknown; M_age_ = 19.1 (18–35), SD = 1.58), who completed either of the two questionnaires. Of the final sample, 390 respondents (65.7%) participated at both T1 and T2, 66 (12.7%) participated only at T1, and 63 (12.3%) participated only at T2. Both cohorts were equally represented in the final sample (2017: *n* = 268; 51.6%, 2018: *n* = 251; 48.4%); no significant differences were found between cohorts on any of the study’s variables.

### Measures

At both T1 and T2, respondents completed the 40-item version of the MPQ-SF [30], consisting of 8 items per dimension, which ask respondents to self-report whether certain personality characteristics are applicable to themselves, on a 7-point Likert Scale (1 = not at all applicable; 7 = completely applicable). Cultural Empathy (CE) was measured using 8 items, such as ‘Pays attention to the emotions of others’; Emotional Stability (ES) was measured using 8 items, such as ‘Keeps calm when things don’t go well’; Flexibility (FX) was measured using 8 items, such as ‘Looks for regularity in life’ (Reversed); Openmindedness (OP) was measured using 8 items, such as ‘Seeks people from different backgrounds’; Social Initiative (SI) was measured using 8 items, such as ‘Is inclined to speak out’.

Respondents were also asked to voluntarily indicate their age (in years), their gender (female / male / other), and their country of birth (open question). The data on country of birth was used as a proxy for the respondents’ cultural background. In total, 74 countries were represented in the sample.

Ideally, a full cross-cultural validation would involve examining invariance between a number of cultural groups, each with sufficient sample size to reliably fit the different models under scrutiny [35]. However, due to limited sample sizes of specific national groups in our sample, the present study makes use of only two aggregated groups, consisting of students of *Western* and *Non-Western* background. The Western group included those who were born in Europe, North America, Australia, or New Zealand (*n =* 283, 54.5%), of which those from the Netherlands (29.1%), Germany (4.6%), France (1.7%), Italy (1.3%) and the United Kingdom (1.2%) were the largest subgroups. The Non-Western group included respondents born elsewhere (*n* = 105; 20.2%), of which those from Vietnam (5.3%), China (2.0%), India (1.7%) and Indonesia (1.0%) were the largest subgroups. For 131 respondents (25.2%), country of birth was unknown.

In Table 1, we provide an overview of the reliability and descriptive statistics of all the MPQ-SF subscales at both time points, as well as their correlations with each other and with control variables.

**Table 1:** Descriptives and correlations of the MPQ-SF dimensions at T1 and T2.

				*T1*					*T2*				
	α	M	SD	CE	ES	FX	OP	SI	CE	ES	FX	OP	SI
*T1*													
CE	.79	5.99	.58	-	0.041	-.106*	.523***	.321***	.706***	.016	-.102	.384***	.213***
ES	.78	4.14	.91		-	.142**	.274***	.228***	-.015	.718***	.114*	.223**	.259**
FX	.78	3.88	.90			-	.048	.016	-.085	.149**	.701***	.080	-.029
OP	.72	5.41	.66				-	.512***	.400***	.182**	.075	.700***	.384***
SI	.82	5.18	.84					-	.227***	.204***	-.001	.429***	.778***
*T2*													
CE	.80	5.82	.63						-	-.024	-.099*	.423***	.223***
ES	.80	3.85	.96							-	.187***	.192***	.260***
FX	.80	3.70	.94								-	.038	.018
OP	.70	5.17	.67									-	.464***
SI	.84	4.94	.90										-
Age (years)		19.10	1.57	.001	.016	.004	.139**	.011	-.074	.031	-.012	.087	.002
Gender (0 = f; 1 = m)				.151**	-.063	-.084	.045	.081	.065	-.070	-.143**	-.049	.062
Cultural Background (0 = West.; 1 = Non-West.)				.002	-.072	.002	.088	-.105*	-.080	-.038	.016	.061	-.081

Note:

* *p* < .05;

** *p* < .01;

*** *p* < .001.

### Analyses

In line with established procedures [36–38], we examined between-group measurement invariance by testing the factor structure across gender (male vs. female) and cultural background (Western vs. Non-Western), through three separate models. First, we fitted a configural invariance model (same number of factors between groups). Next, we fitted a weak invariance model, in which the factor loadings were constrained to be equal between groups. Finally, we fitted a strong invariance model in which, next to the loadings, the intercepts of the items were also constrained to be equal between groups.

To test the successive ‘degrees’ of measurement invariance, we compared the fit of the weak invariance model to that of the configural invariance model, and the fit of the strong invariance model to that of the weak invariance model. We also tested if the configural invariance model had sufficient fit to the data. Significant differences in Chi-square, changes in Comparative Fit Index (CFI) larger than .01, changes in Root Mean Square Error of Approximation (RMSEA) larger than .015, and changes in Standardized Root Mean Square Residual (SRMR) larger than .030 were taken as indication of significantly worse fit between the models [39]. If the weak invariance model fit worse than the configural model, individual loadings were freely estimated between groups, and the number of freely estimated loadings was increased (with additional free loadings added in order of the loadings with the largest difference between groups), until the (now partial) weak invariance model did not fit worse than the configural model. Similarly, if the strong invariance model fitted worse than the weak or partial weak invariance model, individual intercepts were freely estimated across groups (with free intercepts added in order of the intercepts with largest difference between groups) until the (partial) strong invariance model did not fit worse than the weak or partial weak invariance model. This procedure was followed for each of the five factors of the MPQ-SF, for T1 and T2 separately.

Similarly, we tested longitudinal measurement invariance by successively testing configural, weak, and strong invariance, but now across timepoints, freeing individual loadings and/or intercepts if necessary. When testing longitudinal invariance, we allowed the residuals of the indicators to covary between timepoints in all models.

## Results

### Measurement invariance for gender

As shown in Table 2, for Cultural Empathy (CE), a 1 factor model had good fit at T1 and moderate to good fit at T2. At T1 the CE model had partial strong invariance for Gender (with only the intercepts of items 2 and 5 needing to be estimated separately for males and females). At T2 the CE model again showed partial strong invariance for Gender (with only the loading for item 4 needing to be varied across gender).

**Table 2:** Measurement invariance for gender.

Model	*χ*^2^(df)	*p*	CFI	RMSEA	SRMR	Δ*χ*^2^(df)	*p*
T1: CE Configural	54.00 (38)	.044	.978	.047	.039		
T1: CE Weak	61.90 (46)	.059	.978	.042	.053	7.89 (8)	.444
T1: CE Strong	84.25 (54)	.005	.959	.054	.067	22.35 (8)	.004
T1: CE Partial	73.92 (52)	< .001	.970	.047	.061	12.03 (6)	.060
T2: CE Configural	85.05 (38)	< .001	.928	.086	.049		
T2: CE Weak	103.97 (46)	< .001	.911	.086	.068	18.92 (8)	.015
T2: CE Part.Weak	99.01 (45)	< .001	.917	.084	.063	13.96 (7)	.052
T2: CE Partial	103.95 (53)	< .001	.922	.076	.065	4.94 (8)	.764
T1: ES Configural	88.95 (36)	< .001	.929	.087	.050		
T1: ES Weak	93.769 (44)	< .001	.933	.076	.054	4.82 (8)	.777
T1: ES Strong	114.35 (52)	< .001	.916	.079	.061	20.58 (8)	.008
T1: ES Partial	103.41 (50)	< .001	.928	.074	.057	9.64 (6)	.141
T2: ES Configural	61.06 (36)	.006	.966	.064	.043		
T2: ES Weak	72.76 (44)	.004	.961	.062	.066	11.70 (8)	.165
T2: ES Strong	86.61 (52)	.002	.953	.063	.070	13.85 (8)	.086
T1: FX Configural	81.98 (36)	< .001	.936	.081	.051		
T1: FX Weak	87.43 (44)	< .001	.940	.071	.059	5.45 (8)	.708
T1: FX Strong	110.50 (52)	< .001	.919	.076	.069	23.08 (8)	.003
T1: FX Partial	98.03 (50)	< .001	.934	.070	.063	10.61 (6)	.101
T2: FX Configural	98.44 (36)	< .001	.927	.101	.064		
T2: FX Weak	119.74 (44)	< .001	.911	.101	.073	21.30 (8)	.006
T2: FX Part.Weak	112.25 (43)	< .001	.919	.098	.072	13.82 (7)	.055
T2: FX Partial	126.33 (49)	< .001	.910	.097	.080	14.08 (6)	.029
T1: OP Configural	44.46 (40)	.289	.989	.024	.036		
T1: OP Weak	55.77 (48)	.206	.981	.029	.047	11.31 (8)	.185
T1: OP Strong	74.36 (56)	.051	.956	.041	.052	18.59 (8)	.017
T1: OP Partial	66.11 (55)	.145	.973	.032	.049	10.34 (7)	.170
T2: OP Configural	87.80 (38)	< .001	.858	.088	.058		
T2: OP Weak	97.98 (46)	< .001	.852	.082	.067	10.18 (8)	.253
T2: OP Strong	102.54 (54)	< .001	.862	.073	.069	4.57 (8)	.803
T1: SI Configural	133.95 (38)	< .001	.912	.114	.069		
T1: SI Weak	141.85 (46)	< .001	.912	.104	.085	7.90 (8)	.444
T1: SI Strong	153.69 (54)	< .001	.909	.097	.090	11.84 (8)	.158
T2: SI Configural	146.92 (38)	< .001	.897	.130	.072		
T2: SI Weak	177.11 (46)	< .001	.876	.130	.138	30.20 (8)	< .001
T2: SI Part.weak	157.55 (43)	< .001	.891	.126	.106	10.64 (5)	.059
T2: SI Partial	163.65 (51)	< .001	.893	.114	.108	6.03 (8)	.637

Note: Male: *n* = 75; Female: *n* = 314.

For the Emotional Stability (ES) factor, items 2 and 8 and items 4 and 6 were allowed to have residual covariances in order to get sufficient model fit. With these additions the configural model had moderate to good fit at both T1 and T2. At T1 the ES model had partial strong invariance for Gender (with only the intercepts of items 4 and 8 needing to be estimated separately for males and females). At T2 the ES model showed strong invariance for Gender.

For the Flexibility (FX) factor, items 2 and 3 and items 4 and 5 were allowed to have residual covariances in order to get sufficient model fit. With these additions the factor model had moderate to good fit T1 and weak to moderate fit at T2. At T1 the FX model had partial strong invariance for Gender (with only the intercepts for items 1 and 5 estimated freely across groups). At T2 the FX model again showed partial strong invariance for Gender (with the loading for item 4, and the intercepts for items 2 and 3 needing to be varied across groups).

For the Openmindedness (OP) factor, the model did not fit the data at T2 well, but since the longitudinal configural model had good fit (see Table 3 and discussion below) we decided not to add any residual covariances. At T1 the OP model had partial strong invariance for Gender (with only the intercepts for item 7 estimated freely across Gender). At T2, the OP model had strong invariance for Gender.

**Table 3:** Measurement invariance for cultural background.

Model	*χ*^2^(df)	*p*	CFI	RMSEA	SRMR	Δ*χ*^2^(df)	*p*
T1: CE Configural	64.70 (38)	.004	.965	.060	.041		
T1: CE Weak	74.99 (46)	.004	.962	.057	.068	10.28 (8)	.246
T1: CE Strong	83.47 (54)	.006	.962	.053	.069	8.48 (8)	.388
T2: CE Configural	95.21 (38)	< .001	.911	.095	.048		
T2: CE Weak	112.96 (46)	< .001	.896	.093	.083	17.74 (8)	.023
T2: CE Part.Weak	105.76 (45)	< .001	.905	.090	.069	10.55 (7)	.160
T2: CE Partial	112.08 (53)	< .001	.908	.081	.072	6.32 (8)	.611
T1: ES Configural	78.19 (36)	< .001	.943	.078	.049		
T1: ES Weak	88.78 (44)	< .001	.939	.072	.062	10.60 (8)	.226
T1: ES Strong	96.13 (52)	< .001	.940	.066	.066	7.35 (8)	.500
T2: ES Configural	49.76 (36)	.063	.981	.048	.041		
T2: ES Weak	59.75 (44)	.057	.978	.046	.057	9.99 (8)	.266
T2: ES Strong	67.06 (52)	.078	.979	.041	.060	7.31 (8)	.504
T1: FX Configural	84.16 (36)	< .001	.934	.083	.052		
T1: FX Weak	97.79 (44)	< .001	.926	.079	.067	13.63 (8)	.092
T1: FX Strong	117.46 (52)	< .001	.910	.080	.074	19.67 (8)	.012
T1: FX Partial	107.83 (51)	< .001	.922	.076	.071	10.04 (7)	.186
T2: FX Configural	98.72 (36)	< .001	.928	.102	.066		
T2: FX Weak	108.40 (44)	< .001	.926	.093	.092	9.69 (8)	.287
T2: FX Strong	128.45 (52)	< .001	.913	.093	.098	20.04 (8)	.010
T2: FX Partial	121.36 (51)	< .001	.920	.090	.096	12.85 (7)	.073
T1: OP Configural	39.13 (40)	.509	1.000	.000	.034		
T1: OP Weak	48.46 (48)	.454	.999	.007	.052	9.33 (8)	.315
T1: OP Strong	60.56 (56)	.315	.989	.020	.059	12.09 (8)	.147
T2: OP Configural	79.78 (38)	< .001	.878	.081	.061		
T2: OP Weak	91.207 (46)	< .001	.868	.076	.070	11.43 (8)	.179
T2: OP Strong	101.94 (54)	< .001	.860	.073	.075	10.73 (8)	.217
T1: SI Configural	147.74 (38)	< .001	.903	.122	.073		
T1: SI Weak	167.81 (46)	< .001	.892	.117	.096	20.08 (8)	.010
T1: SI Part.Weak	155.79 (45)	< .001	.902	.113	.082	8.05 (7)	.328
T1: SI Partial	173.02 (52)	< .001	.893	.109	.086	17.23 (7)	.016
T2: SI Configural	156.74 (38)	< .001	.887	.136	.072		
T2: SI Weak	163.73 (46)	< .001	.888	.123	.098	6.99 (8)	.538
T2: SI Strong	181.90 (54)	< .001	.879	.119	.103	18.17 (8)	.020
T2: SI Partial	174.69 (53)	< .001	.885	.117	.100	10.96 (7)	.140

Note: Western: *n =* 283; Non-Western: *n* = 105.

For the Social Initiative (SI) factor model, items 3 and 7 needed to be correlated to get weak to moderate fit at both T1 and T2. At T1 the SI model had strong invariance for Gender. At T2 the SI model showed partial strong invariance (with the loadings for items 2, 4, and 5 needing separate estimation across genders).

In sum, for the subscales of the MPQ-SF, we found only a small number of parameters that differed between males and females. Due to the fact that these differences were not constant across time (different parameters needed to be freely estimated at T1 compared to T2), and the number of parameters that needed to be freely estimated was small, we conclude that for all factors, the models could meaningfully be fitted to the sample as a whole. Our findings thus show that the MPQ-SF performs similarly among both males and females.

### Measurement invariance for cultural groups

The same procedure was followed in comparing how the MPQ-SF factors performed among Western vs. Non-Western cultural groups. As shown in Table 3, CE displayed strong invariance for cultural group at T1, and partial strong invariance at T2 (with only the loading for item 7 needing to be freely estimated).

At both T1 and T2, the ES model had strong invariance for cultural group.

At T1 the FX model had partial strong invariance for cultural group (with only the intercepts for item 6 estimated freely across groups). At T2 the FX model also showed partial strong invariance for cultural group (with only the loading for item 5 estimated separately for the Western and non-Western group).

At both T1 and T2, the OP model had strong invariance for cultural group.

At T1 the SI model had partial strong invariance for cultural group (with the loading for item 4, and the intercept for item 8 needing separate estimation across groups). At T2 the SI model showed partial strong invariance for cultural group (with only the loading for item 2 estimated separately for the Western and non-Western group).

For the subscales of the MPQ-SF, we found only a small number of parameters that differed between the Western and Non-Western groups. As before, due to the fact that these differences were not constant across time, and only a small number of parameters needed to be freely estimated, we conclude that for all factors, the models could meaningfully be fitted to the sample as a whole. Our findings show that the MPQ-SF performs similarly among both Western and Non-Western respondents.

### Longitudinal measurement invariance

To examine longitudinal measurement invariance, we tested the performance of the MPQ-SF factors across the two time points. As was explained above, 75.1% (*n* = 390) of the sample participated in the study at both T1 and T2. We first examined invariance using only this subsample. Next, the same models were tested using Full Information Maximum Likelihood (FIML) estimation on the full sample (*n* = 519), thus including participants with missing data on one of the time points. Results were comparable across both sets of analyses.

Table 4 displays the results for our examination of measurement invariance across time points using FIML on the full sample. As is customary in testing for longitudinal measurement invariance, we allowed the residuals of the indicators to covary between timepoints in all models.

**Table 4:** Longitudinal measurement invariance.

Model	*χ*^2^(df)	*p*	CFI	RMSEA	SRMR	Δ*χ*^2^(df)	*p*
CE T1	33.23(19)	.023	.981	.043	.031		
CE T2	67.38(19)	< .001	.944	.075	.040		
CE Configural	220.91(95)	< .001	.944	.051	.046		
CE Weak	243.17(102)	< .001	.934	.052	.070	22.26(7)	.002
CE Strong	274.87(109)	< .001	.926	.055	.085	31.70(7)	< .001
ES T1	53.83(18)	< .001	.951	.070	.043		
ES T2	50.95(18)	< .001	.964	.064	.038		
ES Configural	209.28(91)	< .001	.949	.050	.051		
ES Weak	215.08(98)	< .001	.949	.048	.054	5.80(7)	.564
ES Strong	280.54(105)	< .001	.924	.057	.064	65.46(7)	< .001
ES Part Strong	227.60(104)	< .001	.947	.048	.057	12.525(7)	.051
FX T1	29.04(18)	< .001	.944	.075	.045		
FX T2	118.67(18)	< .001	.902	.111	.065		
FX Configural	274.76(91)	< .001	.922	.063	.060		
FX Weak	285.30(98)	< .001	.920	.061	.062	10.54(7)	.160
FX Strong	345.60(105)	< .001	.898	.067	.065	60.30(7)	< .001
FX Part Strong	300.09(103)	< .001	.916	.061	.062	14.79(5)	.011
OP T1	28.81(20)	.092	.979	.033	.030		
OP T2	65.82(20)	< .001	.894	.071	.045		
OP Configural	159.18(95)	< .001	.957	.036	.041		
OP Weak	160.81(102)	< .001	.961	.034	.043	1.63(7)	.978
OP Strong	165.58(109)	< .001	.962	.032	.044	4.77(7)	.688
SI T1	120.56(19)	< .001	.912	.115	.066		
SI T2	141.27(19)	< .001	.912	.119	.064		
SI Configural	384.17(93)	< .001	.907	.078	.072		
SI Weak	391.34(100)	< .001	.907	.076	.075	7.17(7)	.412
SI Strong	407.59(107)	< .001	.904	.074	.076	16.25(7)	.023

Note: *n* = 519.

For CE, the configural invariance model had moderate to good fit to the data. While the difference in chi-square was significant between the weak and configural model, and between the strong and the weak model for CE, the differences in CFI, RMSEA, and SRMR were smaller than the boundaries specified above for worse model fit. We therefore concluded that the model for CE displayed strong measurement invariance across time.

For the ES factor, the configural model had moderate to good fit to the data. The weak invariance model did not fit significantly worse than the configural model, but the difference in fit between the strong and weak invariance model was too large to conclude strong invariance across time. After allowing the intercept of item 7 to vary across timepoints, the difference with the weak invariance was no longer significant. We therefore conclude that the ES model had partial strong invariance across time, with only the intercept of item 7 differing between timepoints.

For the FX factor, the configural model had moderate to good fit to the data when allowing for these two residual correlations. The weak invariance model did not fit significantly worse than the configural model, but the difference in fit between the strong and weak invariance model was again too large to conclude strong invariance across time. After allowing the intercepts of items 2 and 7 to vary across timepoints we found the difference between the weak invariance and partial strong invariance model to be small enough to conclude partial strong invariance. We therefore concluded that the FX model had partial strong invariance across time, with only the intercepts of items 2 and 7 differing between timepoints.

For the OP factor, because the longitudinal configural model had good fit we decided not to add any residual covariances. The weak model did not fit worse than the configural model and the strong invariance model did not fit worse than the weak invariance model, implying that the model for OP showed strong measurement invariance across time.

For the SI factor, the configural invariance model displayed moderate fit. Because we wanted to stay as close to the theoretical models as possible, we decided to not add any more residual correlations and use the moderate fitting longitudinal configural model as our starting point. As the weak invariance model did not fit less well than the configural model (following the rules specified above), the strong invariance model did not fit less well than the weak invariance model, and since the strong invariance had moderate fit to the data (despite the configural model also only having moderate fit) we concluded that the model for SI showed strong invariance across time.

Overall, our results show that the subscales of the MPQ-SF display sufficient measurement invariance across time points to be reliably used in longitudinal research.

## Discussion

### Summary of findings and implications

The question how intercultural competences, and specifically multicultural personality traits, develop in international training and education has become a major area of interest in intercultural research [1]. Understanding the development of such complex constructs over time requires the use of reliable, yet practical measures, of which not many are available. A promising instrument for measuring multicultural personality development is the 40-item short form of the Multicultural Personality Questionnaire (MPQ-SF), which has previously been established as a reliable alternative to the long version of the instrument [12, 30]. However, further validation of the MPQ-SF was needed among different groups of respondents. Although the long version of the MPQ has already been shown to be reliable in different samples [12, 40], this is the first study to examine measurement invariance between different groups of respondents for the short form.

Our results show that all five subscales perform similarly among both male and female students, as well as among Western and Non-Western students. Based on our findings, we can conclude that the MPQ-SF can be reliably used to measure and compare multicultural personality within and across these groups. The fact that the scales appear to be reliable among respondents with different cultural backgrounds is particularly important, because the instrument is specifically intended to be used in intercultural contexts. The second aim of this study was to examine longitudinal measurement invariance, by comparing how the MPQ-SF performs at two different time points, five months apart. Again, our results show sufficient invariance between these time points, and we conclude that the MPQ-SF can be reliably used in longitudinal research in the context of international education.

This opens up many new opportunities for further exploration, allowing scholars to track changes in respondents’ multicultural personality over time, and examine factors which may influence how individuals’ scores on the five subscales develop as a result of training or studying in an intercultural university program. Particularly in the context of international education, such studies may reveal new insights in the possibility to increase intercultural competences of students and staff. For example, it can be used to enhance the effects of going on an international exchange, to maximize the effects of cultural diversity in the students’ social/educational environment, or by increasing interaction between local and international students.

At this point, a growing research tradition based on the MPQ and MPQ-SF will allow us to get a deeper understanding of these issues, which is crucial in informing institutional management on how to design new policies towards internationalization of higher education, and how to aid educators in designing a more inclusive multicultural classroom.

### Limitations

As with any research, the present study has several limitations. The most notable is that, as with the original study that was used to develop the MPQ-SF, the present research was conducted using a sample of university students. Although this is an interesting and relevant respondent group, particularly considering the possible uses of the MPQ-SF in the context of higher education, it does limit generalizability. The original MPQ has already been validated among different groups of respondents, such as expatriates [24], migrants [14], participants in intercultural training [15] and job applicants [25]. We recommend future scholars who use the MPQ-SF in such contexts, to also test for measurement invariance in their own sample.

The second limitation of the present study is that our cross-cultural validation relies on comparison between two respondent groups only. A full cross-cultural validation examines cultural bias in item responses, between a larger number of cultural groups, each with sufficient sample size to reliably fit the different models under scrutiny [35]. However, due to limited sample sizes of specific national groups in our sample, the present study makes use of only two aggregated groups, consisting of Western and Non-Western respondents. As such, our findings provide sufficient support for reliable use of the MPQ-SF in a diverse intercultural context, such as higher education. However, for directly comparing levels of multicultural personality between specific cultural groups, a more detailed cross-cultural validation should be conducted first.

Finally, a word of caution for those who are interested in applying the MPQ in a professional context, for example in diagnostics or assessment. The use of short form measures to assess personality traits has a number of limitations that one should be aware of [41]. Although many of the possible deficiencies of the short-form have been mitigated through this study and earlier work [30], the full version of the MPQ remains the instrument of choice for such settings.

## Conclusion

The MPQ-SF is shown to be a useful and reliable instrument for measuring multicultural personality in research contexts where time or survey space is limited, or where drop-out of respondents more likely to occur. Through a study conducted in an international university context, we have established that all five subscales perform similarly among men and women, as well as among Western and Non-Western respondents, and that the instrument is invariant across time points. This allows the instrument to be used in studying the longitudinal effects of international education on multicultural personality.
